# Description of Aeminiaceae fam. nov., *Aeminium* gen. nov. and *Aeminiumludgeri* sp. nov. (Capnodiales), isolated from a biodeteriorated art-piece in the Old Cathedral of Coimbra, Portugal

**DOI:** 10.3897/mycokeys.45.31799

**Published:** 2019-01-28

**Authors:** João Trovão, Igor Tiago, Fabiana Soares, Diana Sofia Paiva, Nuno Mesquita, Catarina Coelho, Lídia Catarino, Francisco Gil, António Portugal

**Affiliations:** 1 Centre for Functional Ecology, Science for People and the Planet, University of Coimbra, Coimbra, Portugal University of Coimbra Coimbra Portugal; 2 Laboratory for Plant Health (Fitolab), Instituto Pedro Nunes, Coimbra, Portugal Instituto Pedro Nunes Coimbra Portugal; 3 Geosciences Center, University of Coimbra, Coimbra, Portugal University of Coimbra Coimbra Portugal; 4 Center for Physics of the University of Coimbra (CfisUC), Coimbra, Portugal Instituto Pedro Nunes Coimbra Portugal

**Keywords:** Biodeterioration, Capnodiales, microcolonial black fungi, phylogeny, taxonomy

## Abstract

When colonizing stone monuments, microcolonial black fungi are considered one of the most severe and resistant groups of biodeteriorating organisms, posing a very difficult challenge to conservators and biologists working with cultural heritage preservation. During an experimental survey aimed to isolate fungi from a biodeteriorated limestone art piece in the Old Cathedral of Coimbra, Portugal (a UNESCO World Heritage Site), an unknown microcolonial black fungus was retrieved. The isolated fungus was studied through a complete examination based on multilocus phylogeny of a combined dataset of ITS rDNA, LSU and *rpb2*, in conjunction with morphological, physiological, and ecological characteristics. This integrative analysis allows for the description of a new family, Aeminiaceae fam. nov., a new genus *Aeminium* gen. nov., and a new species, *Aeminiumludgeri* sp. nov., in the order Capnodiales.

## Introduction

Microcolonial black fungi (MCBF) are a remarkably diverse fungal group characterized by unique phenotypic features, such as strongly melanized cell walls, slow growth, ability to shift from a mycelial to a meristematic state, high morphological plasticity, and predominant asexual reproduction (Butinar et al. 2005, Sterflinger 2006, Selbmann et al. 2015). They exhibit several physiological adaptations allowing their tolerance to various stress factors, including extreme temperatures, high solar and ultraviolet radiation, osmotic changes, and severe drought (Sterflinger 2006; Zakharova et al. 2013, Selbmann et al. 2015). This set of unique characteristics results from their adaptation to oligotrophic lifestyles, which are achieved through the production of protective molecules such as mycosporines and carotenoids (Gorbushina et al. 2003, 2008), restricted compacted growth, predominant specialized survival structures, and simple life cycles (Selbmann et al. 2005). Understandably, the ecology of MCBF reflects their resistance, as they occur in extreme environments such as hot and cold deserts, saltpans, acidic and hydrocarbon-contaminated sites, and exposed rocks surfaces (Selbmann et al. 2015). When colonizing stone monuments and art pieces, they deepen fissures and cracks through hyphal penetration (biopitting) (Sterflinger and Krumbein 1997; Lombardozzi et al. 2012) and the production of corrosive extracellular polysaccharides (Sterflinger 2006; Selbmann et al. 2014), synergistically promoting aesthetic, biophysic, and biochemical dismantlement of the material. Due to their powerful destructive potential and their high resistance to many types of restoration treatments, they are one of the major challenges for conservators and biologists working with biodeterioration of cultural heritage materials (Isola et al. 2013).

Classical morphological approaches used to identify MCBF are largely inefficient due to their extremely poor differentiation and, in some cases, to the existence of polymorphic traits (Sterflinger 2006). Phylogenetic analyses have revealed that their ability to grow on rock substrates is a polyphyletic trait and these organisms are mainly classified in class Dothideomycetes (orders Capnodiales, Dothideales, and Pleosporales) and class Eurotiomycetes (order Chaetotyriales) (Selbmann et al. 2013, 2014). Phylogenetic studies of MCBF belonging to order Capnodiales (collected by Friedmann (1982), Selbmann (2005, 2008), Ruibal et al. (2005, 2009, 2011) and Egidi et al. (2014)), have shown that several genera of slow-growing MCBF belonged to family Teratosphaeriaceae and/or to closely associated and unclassified families, initially referred to as Teratosphaeriaceae “1” and “2”. These two families were further resolved by Quaedvlieg et al. (2014) by applying the consolidated species concept (CSC) to circumcise the majority of these organisms in two novel families, Neodevriesiaceae and Extremaceae. Neodevriesiaceae and Extremaceae were further arranged and expanded by Crous et al. (2015), Isola et al. (2016), Wang et al. (2017), and Delgado et al. (2018), deepening the available knowledge of fungi in this order.

In 2013, UNESCO recognized the University of Coimbra, Alta and Sofia (Coimbra, Portugal) as a World Heritage Site. Inside this area, several monuments exhibit clear signs of biodeterioration, including microcolonial black fungi proliferation. During one experimental survey performed in the Old Cathedral of Coimbra (Sé Velha de Coimbra), an unknown slow-growing microcolonial black fungi with late melanization was retrieved. Therefore, we aim to determine, through a multi-gene analysis (ITS rDNA, LSU and *rpb2*) coupled with a morphological, physiological and ecological examination, the taxonomic status and position of this fungus in the order Capnodiales.

## Materials and methods

### Site description, sample collection and fungal isolation

The Old Cathedral of Coimbra is the only Portuguese Romanesque cathedral from the Reconquista times to have survived relatively intact until now. The Romanesque church is located on a hillside in the historic city center and was constructed between the 12^th^ and early 13^th^ centuries. The single-floored cloister is arranged laterally to the south of the church and is surrounded by five chapels carved in yellow dolomitic limestone. Samples were collected using sterile scalpels by scrapping small areas (3 cm^2^) into a collection tube, from a deteriorated art-piece in the Santa Maria chapel (40°12'32"N, 8°25'38"W) (Suppl. material 1: Figure S1). All sampling procedures were performed with the permission of the local government authority (Direcção Regional de Cultura do Centro) and supervised by technicians from the cathedral. From the 10 retrieved isolates, one was obtained through the suspension of the retrieved sample in 1.5 ml of sterile 0.9% (w/v) NaCl solution, vortexing and plating over Malt Extract Agar (MEA) (Difco, USA) supplemented with NaCl (10%) and streptomycin (0.5 g L^–1^) and nine originated by the spread plate technique on solid DSMZ 372- Halobacteria medium (DSMZ, Germany), NaCl 20% (w/v), pH 8, of an aliquot of a 7 days enrichment culture obtained in the same liquid medium. Inoculated plates were incubated aerobically, in the dark at room temperature (28±1 °C) and the different colonies were isolated to axenic cultures in Potato Dextrose Agar medium (PDA), (Difco, USA).

### DNA extraction, PCR amplification and sequencing

DNA from pure fungal cultures was obtained using the Extract-N-Amp Plant PCR Kit (Sigma-Aldrich, USA) with several modifications. A small portion of the colonies was scraped from the agar surface using a sterile scalpel, submerged in 10 µl of extraction solution and incubated in an ABI GeneAmp 9700 PCR System (Applied Biosystems, USA), with the following protocol: 65 °C for 10 min, followed by 95 °C for 15 min. After the incubation, reactions were stopped by adding 10 µl of elution solution. The obtained genomic DNA was subjected to PCR amplification with a final volume of 25 µl, with 12.5 µl of NZYTaq Green Master Mix (NZYTech, Portugal), 1 µl of each primer (10 mM), 9.5 µl of ultra-pure water and 1 µl of template DNA. Primer pairs ITS1-F/ITS4 (White et al. 1990; Gardes and Bruns 1993), LSU1fd/LR5 (Vilgalys and Hester 1990; Crous et al. 2009) and f*rpb2*-5F/f*rpb2*-414R (Liu et al. 1999; Quaedvlieg et al. 2011) were used to amplify the ITS, LSU and *rpb2* regions, respectively. PCR reactions were performed using an ABI GeneAmp 9700 PCR System (Applied Biosystems, USA), with the following conditions: initial denaturation temperature of 96 °C for 2 min, followed by 40 cycles of denaturation temperature of 96 °C for 45 s, primer annealing at 54 °C (ITS), 52 °C (LSU), 49 °C (*rpb2*), primer extension at 72 °C for 90 s, and a final extension step at 72 °C for 2 min. Obtained amplicons were purified using the NZYGelpure DNA purification kit (NZYTech, Portugal) and sequenced using an ABI 3730xl DNA Analyzer system (96 capillary instruments) using the BigDye v. 3.1 Terminator Cycle Sequencing Ready Reaction Kit (Applied Biosystems, USA) at GATC-Biotech, Germany.

### Phylogenetic analysis

DNA sequences were assembled using the Geneious R11.0.02 software (https://www.geneious.com), deposited in GenBank and compared with sequences from the National Center of Biotechnology Information nucleotide databases using NCBIs Basic Local Alignment Search Tool (BLAST), with the option Standard nucleotide BLAST of BLASTN v. 2.6 (Altschul et al. 1997). For construction of the datasets, additional representative sequences of the different families of the order Capnodiales were retrieved from GenBank based on the studies of Quaedvlieg et al. (2014), Isola et al. (2016), Wang et al. (2017), Delgado et al. (2018), (Suppl. material 2: Table S1). Because the ITS region BLAST analysis was the only gene providing a reasonable match (> 95%) with six environmental sequences obtained from a biodeteriorated limestone studied by Vázquez-Nion and colleagues (2016) in Spain, these sequences were also included in the final analysis (best scores for LSU: 95% *Devriesia* sp. ZWY45 (KP010375.1) and *Devriesia* sp. ZWY38 (KP010374.1); best score for *rpb2*: 86% *Hortaeathailandica* CBS 125423 (KF902206.1)). Sequences of each gene were individually aligned using ClustalX^2^ (Larkin et al. 2007) and manually adjusted using UGENE v. 1.26.3 (Okonechnikov et al. 2012). The resulting individual alignments were concatenated using SeaView v. 4 (Gouy et al. 2010). Prior to the phylogenetic analysis, the model of nucleotide substitution for each individual partition was estimated using TOPALi v. 2.5 (Milne et al. 2009) under the Akaike Information Criterion (AIC). In all partitions the best-fit model was determined to be GTR+I+G. A Bayesian Markov Chain Monte Carlo (MCMC) analysis was performed with MrBayes v. 3.2.6 (Ronquist et al. 2012), with four runs over an initial number of 10 million generations. Trees were saved after each 100 generations and the MCMC heated chain “temperature” was set to the value of 0.15. The run was set to stop automatically when the average standard deviation of split frequencies fell below 0.01. After the analysis has stopped, the distribution of log-likelihood scores was confirmed with the Tracer v. 1.5 software (Rambaut and Drummond 2007) to ensure that the stationary phase and convergence in the analysis had been reached. The sampled topologies below the asymptote (25%) were rejected as part of the burn-in phase and the lasting trees were used to calculate the Bayesian posterior probabilities (BP) in an 50% majority rule consensus tree. The resulting phylogenetic tree was viewed in FigTree v. 1.2.2 (Rambaut and Drummond 2008). The obtained alignment and respective phylogenetic tree were deposited in TreeBASE with the submission ID 23164.

### Physiological analysis

To examine heat resistance, mycelia from grown cultures on PDA were homogenized and heated at 75 °C for 30 min, in a shaking water bath. A small aliquot of the heated suspension was plated on fresh PDA culture medium and examined periodically to evaluate fungal growth (according to Seifert et al. 2004). Colonies were considered heat resistant if growth was observed after a period of 3 months after exposure to the protocol described. To determine NaCl tolerance, strains were cultivated on Malt Extract Agar supplemented with NaCl at different concentrations (5, 10, 15, 20, 25, 30% [w/v]) (adapted from Sterflinger 1998). To determine pH tolerance, strains were cultivated on MEA with pH adjustments from 5 to 11, according to Tiago et al. (2004). In all cases, diameters of the colonies were assessed by measuring two perpendicular diameters per colony, weekly during 4 weeks. For all tests, each case study was evaluated in triplicate.

### Morphological analysis

For morphological characterization, strains were cultivated on PDA (Difco, USA), Malt Extract Agar (MEA), (Difco, USA) with 10% NaCl (w/v) and Dichloran Glycerol Agar (DG-18), (Sigma-Aldrich, USA) for up to 6 months. Morphological analysis was performed directly on the cultured media plates or using the slide culture technique. Preparations were transferred into slides, observed with a light microscope (Leica DM 4000B (Leica, Germany)), and photographed (Leica DFC 490 digital camera (Leica, Germany)). At least 30 measurements per structure were considered. Representative drawings of microscopic morphological characteristics were obtained with Adobe Illustrator CC (Adobe, USA).

## Results

### Phylogenetic analysis

**Figure 1.**
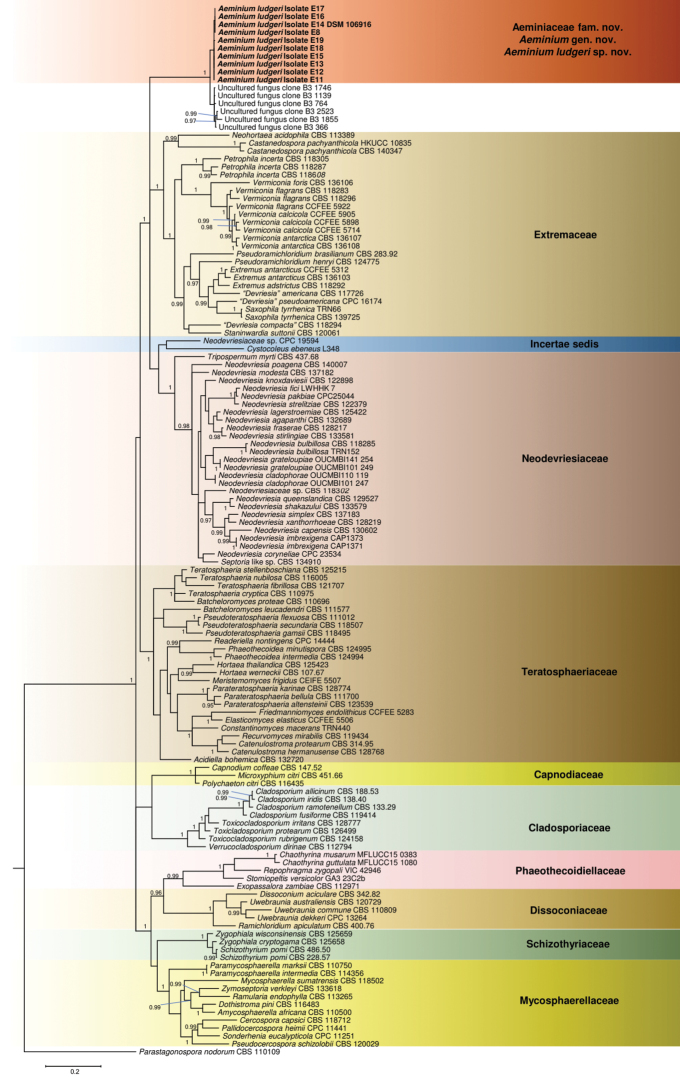
Bayesian 50% majority rule consensus tree based on an LSU/*rpb2*/ITS concatenated alignment, containing representative sequences from the order Capnodiales. The new strains are shown in bold. Bayesian posterior probabilities (BP) ≥ 0.95 are presented at the nodes. The tree was rooted to *Parastagonosporanodorum* CBS 110109. The scale bar specifies 0.2 expected changes per site.

The phylogenetic analysis was performed using the aligned sequences of the concatenated three-gene dataset with 1301 characters (627 for LSU, 204 for *rpb2* and 470 for ITS), encompassing 133 representative sequences belonging to the different families of the order Capnodiales (Fig. 1). From the obtained gene alignments, we were able to verify 260, 143 and 304 unique patterns present in the LSU, *rpb2* and ITS partitions respectively. The MCMC analysis of the three concatenated genes run for 3430000 generations, resulting in 137204 trees. The initial 34300 trees, representative of the analysis burn-in phase was discarded, while the remaining trees were used to calculate posterior probabilities in the majority rule consensus tree. From the phylogenetic data obtained in this study, we were able to verify that the isolated fungi clustered in a monophyletic group with strong support (100% Bayesian posterior probability), distinctly placed from other families in the order Capnodiales but related to the families Extremaceae and Neodevriesiaceae. Thus, this novel lineage is proposed here as a new family Aeminiaceae fam. nov., a new genus, *Aeminium* gen. nov., and a new species *Aeminiumludgeri* sp. nov.

### Physiological studies

Preliminary physiological analysis comprised all the isolates obtained in this study (data not shown). However, as no significant statistical difference was observed among the isolates under the different tested conditions, the final analysis consisted only of data regarding a copy of the culture DSM 106916. No growth was observed for the fungus after exposure to the heat tolerance protocol and therefore, it was classified as non-heat tolerant and non-heat activated. Results for NaCl tolerance test are shown in Suppl. material 3: Figure S2. The fungus was able to grow in NaCl concentrations up to 20% NaCl, with optimal growth occurring at 10% NaCl concentration. No growth was observed for 25% and 30% NaCl concentrations. Nonetheless, the fungus was considered halotolerant, due to the ability to grow in various NaCl concentrations. Results for the pH tolerance test are shown in Suppl. material 4: Figure S3. The fungus showed optimal growth at pH 7 and 9; equal growth values for pH 6 and 8; and no growth was registered for pH 5, 10 and 11. Due to unnecessary pH adjustments for proliferation, strains were considered facultative alkaliphiles. Due to the ability to grown on DG-18 culture media, the fungus was also considered xerophilic.

## Morphological studies

### Taxonomy

#### Aeminiaceae

Taxon classificationFungiCapnodialesAeminiaceae

J. Trovão, I. Tiago & A. Portugal fam. nov.

824975

##### Description.

Asexual morph: mycelium consisting of septate, smooth hyphae, gradually becoming widen, thick-walled, darker and developing into meristematic chains of conidia. Conidia dark brown, thick-walled, smooth, rugose, globose with single central septa resulting from the differentiation of toruloid-like hyphal cells. Sexual morph: unknown.

##### Type genus.

*Aeminium* J. Trovão, I. Tiago & A. Portugal.

##### Type species.

*Aeminiumludgeri* J. Trovão, I. Tiago & A. Portugal.

##### Notes.

Members of Aeminiaceae encompass microcolonial black fungi occurring in deteriorated limestones and are classified as halotolerant, xerophilic, and facultative alkaliphiles. They exhibit slow growth and late melanization, derived from the late differentiation of intercalary or terminal hyphal cells into arthroconidia, that turn olivaceous brown to dark. Fully maturation of the arthroconidia occurs after at least a 2-month incubation period. Their geographical distribution seems to be confined, for now, to limestones in the Iberian Peninsula, although further sampling is necessary to fully highlight their complete geographical and ecological spectrum.

#### Aeminium

Taxon classificationFungiCapnodialesAeminiaceae

J. Trovão, I. Tiago & A. Portugal gen. nov.

824976

##### Description.

Asexual morph: mycelium consisting of septate, smooth hyphae, gradually becoming widen, thick-walled, darker and developing into meristematic chains of conidia. Conidia dark brown, thick-walled, smooth, rugose, globose with single central septa resulting from the differentiation of toruloid-like hyphal cells. Chlamydospores not observed in culture. Sexual morph: unknown.

##### Etymology.

Named after the old Latin name of Coimbra (Aeminium), the city where the strains were isolated.

##### Type species.

*Aeminiumludgeri* J. Trovão, I. Tiago & A. Portugal.

#### Aeminium ludgeri

Taxon classificationFungiCapnodialesAeminiaceae

J. Trovão, I. Tiago & A. Portugal sp. nov.

824977

**Figure 2.**
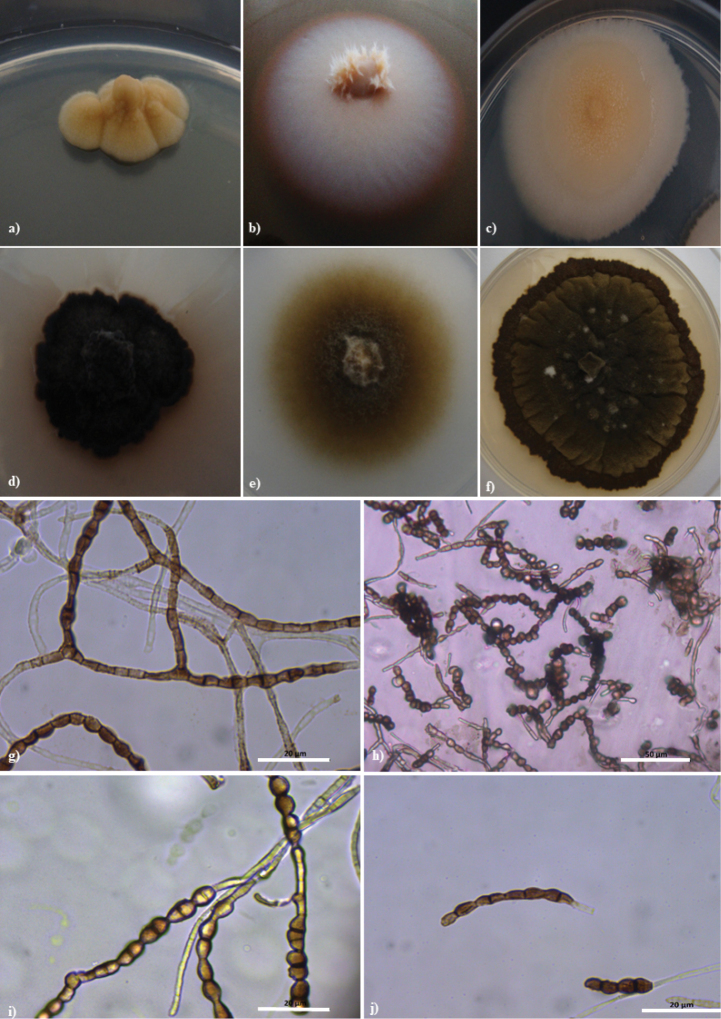
*Aeminiumludgeri***a** Colony appearance on PDA **b** Colony appearance on MEA+10% NaCl (w/v) **c** Colony appearance on DG-18 **d** Colony appearance on PDA after maturation **e** Colony appearance on MEA+10% NaCl (w/v) after maturation **f** Colony appearance on DG-18 after maturation **g** Initial simple, branched, septate hyphae becoming toruloid-like (scale 20 μm) **h** Differentiated, toruloid-like hyphae and mature chains of arthroconidia (scale 50 μm) **i** Intercalary and terminal conidial chains (scale 20 μm); **j** typical aspect of arthroconidia (scale 20 μm)

Figure 2 and Suppl. material 5: Figure S4.

##### Type.

Portugal, Coimbra (40°12'32"N, 8°25'38"W), isolated from a biodeteriorated limestone art piece in the Old Cathedral of Coimbra, 22 November 2016, Igor Tiago, (holotype: permanently preserved in metabolically inactive state DSM 106916).

##### Etymology.

In memory of our late colleague Ludgero Avelar.

##### Diagnosis.

Phylogenetic analysis based on the concatenated ITS rDNA, LSU and *rpb2* dataset considered in the present study clustered the retrieved strains in a monophyletic separate lineage related to the families Neodevriesiaceae and Extremaceae. Therefore, a new family Aeminiaceae fam. nov, a new genus *Aeminium* gen. nov., and a new species *Aeminiumludgeri* sp. nov. in the order Capnodiales are here proposed.

##### Description.

Mycelium initially consisting of branched, septate, smooth, subhyaline to pale green, 2–3 μm wide hyphae. Hyphae moniliform, gradually becoming widen, thick-walled, darker and developing into meristematic conidial chains. Conidiophores micronematous. Arthroconidia dark brown, thick-walled, smooth, sometimes rugose, globose, measuring 3.5–6 × 4.5–6 μm, single central septa, resulting from the differentiation of intercalary or terminal toruloid-like hyphal cells. Sexual morph unknown.

##### Culture characteristics.

On 6 weeks old PDA plates, colonies growing slowly, to 8 mm in diameter, cerebriform, irregular, raised centrally, often moist, deeply immersed into agar, pale pink, with scarce velvety, pale pink-hyaline, aerial, short hyphae and well-defined, small, white, and glossy margin; pale pink on reverse. After at least 2 months, colonies become fully mature and melanized, olivaceous brown-black on top and on reverse. On 6 weeks old MEA+10% NaCl plates, colonies growing slowly, to 25 mm in diameter, flat, circular, moist, pale pink, with prominent velvety pale pink-hyaline aerial short hyphae and with undulate margin; pale pink in reverse. After at least 2 months, colonies become fully mature and melanized, with velvety grey-white aerial short hyphae, filiform margin, olivaceous brown on top and in reverse. On 6-weeks-old DG-18 plates, colonies growing slowly, to 20 mm in diameter, flat, irregular, often moist, deeply immersed into agar, with velvety yellow-pale brown aerial short hyphae and with irregular undulate white margin; yellow-pale brown in reverse. After at least 2 months, colonies become fully mature and melanized, raised, rugose, olivaceous brown-black on top and in reverse.

##### Distribution.

Portugal.

##### Additional specimens examined.

Portugal, Coimbra (40°12'32"N, 8°25'38"W), isolated from a biodeteriorated limestone art piece in the Old Cathedral of Coimbra, I. Tiago, living culture E8, ibid. living culture E11, ibid. living culture E12, ibid. living culture E13, ibid. living culture E14, ibid. living culture E15, ibid. living culture E16, ibid. living culture E17, ibid. living culture E18; ibid. living culture E19.

## Discussion

Here we describe *Aeminiumludgeri*, a new MCBF species, as well as establish a new genus and family within the order Capnodiales to accommodate this fungus. Phylogenetic analyses, based on ITS rDNA, LSU and *rpb2* molecular data, revealed that the retrieved isolates cluster in a separate lineage strongly supported at a family-level, related to the families Extremaceae and Neodevriesiaceae. Although some molecular variance can be observed among the studied isolates, when dealing with MCBF, larger degrees of sequence heterogeneity for species delimitation are accepted due to the lack of sexual recombination, predominance of clonality, and perpetuation of super-adapted genotypes (Egidi et al. 2014). Additionally, due to ITS sequence heterogeneity among the retrieved strains being 1%, a sole new genus and species were considered in Aeminiaceae.

The clustering of the environmental sequences obtained by Vázquez-Nion (2016) with the sequences obtained during this study provides valuable information on the ecology, habitat, and geographical distribution of the new family. So far, Aeminiaceae has only been identified on deteriorated limestones monuments on the Iberian Peninsula. This ecological characteristic is clearly distinct when compared to the related Extremaceae (“epiphyllous, endophytic, saprobic or plant pathogenic”) and Neodevriesiaceae (“foliicolous, saprobic or plant pathogenic”) (Quaedvlieg et al. 2014).

The limestones used in the construction of the Old Cathedral of Coimbra hail from unique areas of Portugal (namely Ançã and Portunhos, near Coimbra), and similar stone structures were exported and used on several “Our Ladies of the O” statues and in the portal of the Royal Hospital in Santiago de Compostela (Spain). We hypothesize that the transportation of limestone to such places might have contributed to the dispersion of this organism and, thus, explain the detection of Aeminiaceae environmental clones in the Cathedral of Santiago de Compostela by Vázquez-Nion et al. (2016). Nonetheless, we also consider that this fungus might be endemic to limestone quarries on the Iberian Peninsula, although acknowledging that additional sampling may further expand the full geographical and ecological spectrum of this fungus.

Physiological tests allowed us to characterize the retrieved isolates as non-heat tolerant, non-heat activated, xerophilic, halotolerant (enduring NaCl concentrations up to 20%), and facultative alkaliphiles. Ecologically, these traits are somewhat more similar to those of Extremaceae (except for pH values, as Aeminiaceae is an alkaliphile and Extremaceae is an acidophile). Regarding heat-tolerance, the isolates are somewhat more similar to Neodevriesiaceae because they do not exhibit heat tolerance. To the best of our knowledge, data regarding heat tolerance for Extremaceae is scarce and could not be further compared. Furthermore, information regarding NaCl tolerance is still poorly described for both Extremaceae and Neodevriesiaceae, but future studies related to stress tolerance in these organisms could provide a valuable character in strain typification.

Although MCBF morphology-based distinction is particularly difficult to accomplish, it can be easily verified that Aeminiaceae differs from the closely related Extremaceae and Neodevriesiaceae due to the necessary period for conidia maturation. Additionally, the verified globose arthroconidia, with single central septa, are distinct from the “subcylindrical to narrowly fusoid-ellipsoidal or obclavate conidia with rarely 1–2 transverse septa” described for Extremaceae and the “rarely septate, solitary conidia composed of a central stalk and two lateral arms with 1–2 transverse septa”, when compared to Neodevriesiaceae (Quaedvlieg et al. 2014). In the five known *Neodevriesia*MCBF species (*N.bulbillosa*, *N.imbrexigena*, *N.modesta*, *N.sardiniae*, and *N.simplex*), production of reproductive cells may occur both through budding (e.g. *N.simplex*) or through meristematic growth (*N.sardiniae*). When considering *A.ludgeri*, only meristematic growth was observed and the new species can be easily distinguished from *N.sardiniae* by the type and much smaller conidia dimensions. Furthermore, no exuded pigments in the agar could be detected in contrast with *N.modesta*, and no chlamydospores were noticed as found in *N.imbrexigena*. When compared with the seven known ExtremaceaeMCBF species (*Extremusadstrictus*, *E.antarcticus*, *Saxophilatyrrhenica*, *Petrophilaincerta*, *Vermiconiaforis*, *V.flagrans*, and *V.antarctica*), *A.ludgeri* is distinctive from *E.antarcticus* and *V.flagrans* by the presence of arthroconidia, and from *V.foris* due to the absence of holoblastic reproductive structures. Moreover, *A.ludgeri* exhibits arthroconidia emerging from meristematic development similar to *E.adstrictus*, *S.tyrrhenica*, *P.incerta*, and *V.antarctica*. Arthroconidia from *A.ludgeri* are, however, clearly distinct from *E.adstrictus* catenate, ellipsoidal conidia, *S.tyrrhenica* thallic-arthric conidia, and *P.incerta* pyriform/ovoidal ramoconidia.

Regarding stone monuments exposed to the environment, microcolonial black fungi are one of the main culprits of stone biodeterioration and are responsible for severe aesthetic, biochemical, and biophysical alterations (Sterflinger 2000, 2010; Sterflinger and Piñar 2013). It is, therefore, crucial to gather a deeper knowledge of the biodiversity of MCBF, and their biological, ecological, and physiological unique characteristics, to allow the development and improvement of tools to protect stone monuments from deterioration.

## Supplementary Material

XML Treatment for Aeminiaceae

XML Treatment for Aeminium

XML Treatment for Aeminium ludgeri

## Supplementary materials

Supplementary material 1 Figure S1. Sampling site Data type: species data Explanation note: a) Cloister of the Old Cathedral of Coimbra; b) lateral view of the Santa Maria Chapel; c) Particular art-piece from where the studied fungi were retrieved (photos by Miguel Mesquita) This is an open access article distributed under the terms of the Creative Commons Attribution License (CC BY 4.0), which permits unrestricted use, distribution, and reproduction in any medium, provided the original author and source are credited.João Trovão, Igor Tiago, Fabiana Soares, Diana Sofia Paiva, Nuno Mesquita, Catarina Coelho, Lídia Catarino, Francisco Gil, António Portugal

Supplementary material 2 Table S1. Fungal strains used in the phylogenetic analysis Data type: phylogenetic data This is an open access article distributed under the terms of the Creative Commons Attribution License (CC BY 4.0), which permits unrestricted use, distribution, and reproduction in any medium, provided the original author and source are credited.João Trovão, Igor Tiago, Fabiana Soares, Diana Sofia Paiva, Nuno Mesquita, Catarina Coelho, Lídia Catarino, Francisco Gil, António Portugal

Supplementary material 3 Figure S2. Growth of *Aeminiumludgeri* incubated at different NaCl concentrations after 4 weeks Data type: statistical data This is an open access article distributed under the terms of the Creative Commons Attribution License (CC BY 4.0), which permits unrestricted use, distribution, and reproduction in any medium, provided the original author and source are credited.João Trovão, Igor Tiago, Fabiana Soares, Diana Sofia Paiva, Nuno Mesquita, Catarina Coelho, Lídia Catarino, Francisco Gil, António Portugal

Supplementary material 4 Figure S3. Growth of *Aeminiumludgeri* incubated at different pHs levels after 4 weeks Data type: statistical data This is an open access article distributed under the terms of the Creative Commons Attribution License (CC BY 4.0), which permits unrestricted use, distribution, and reproduction in any medium, provided the original author and source are credited.João Trovão, Igor Tiago, Fabiana Soares, Diana Sofia Paiva, Nuno Mesquita, Catarina Coelho, Lídia Catarino, Francisco Gil, António Portugal

Supplementary material 5 Figure S4. Representative drawing of *Aeminiumludgeri* Data type: species data Explanation note: a) initial simple, branched, septate hyphae becoming toruloid-like and strongly melanized; b) differentiated, toruloid-like hyphae with chains of arthroconidia; c) and d) arthroconidia (scale 10 μm). This is an open access article distributed under the terms of the Creative Commons Attribution License (CC BY 4.0), which permits unrestricted use, distribution, and reproduction in any medium, provided the original author and source are credited.João Trovão, Igor Tiago, Fabiana Soares, Diana Sofia Paiva, Nuno Mesquita, Catarina Coelho, Lídia Catarino, Francisco Gil, António Portugal
