# Supplementary figures and images for: Description of Aeminiaceae fam. nov., Aeminium gen. nov. and Aeminiumludgeri sp. nov. (Capnodiales), isolated from a biodeteriorated art-piece in the Old Cathedral of Coimbra, Portugal
Source: MycoKeys. 2019 Jan 28;(45):57–73. doi: 10.3897/mycokeys.45.31799 (PMC6361875; doi:10.3897/mycokeys.45.31799)

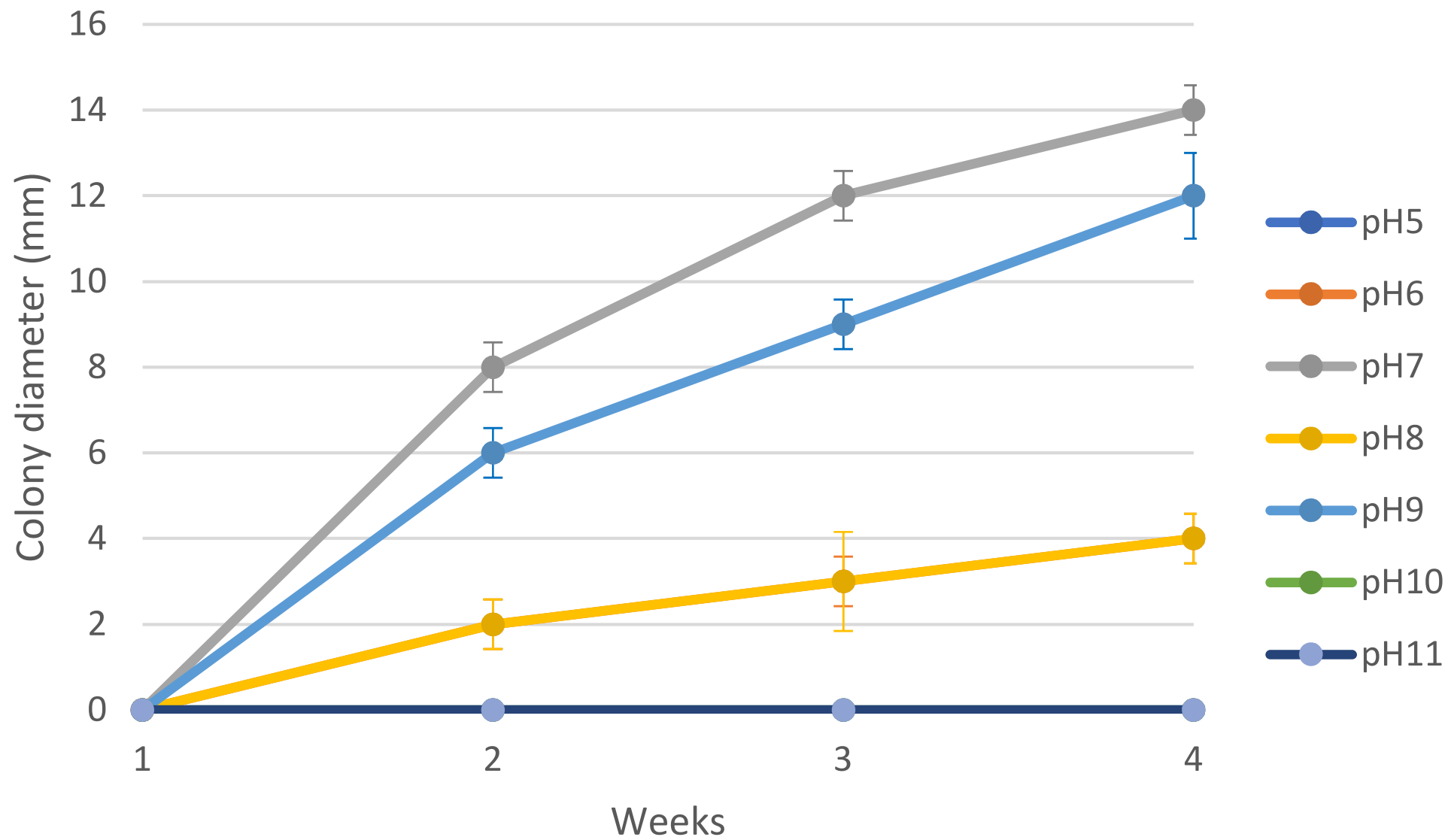

Supplement: Supplementary material 1 [file mycokeys-45-057-s005.pdf]
